# Implementation considerations for a point-of-care *Neisseria gonorrhoeae* rapid diagnostic test at primary healthcare level in South Africa: a qualitative study

**DOI:** 10.1186/s12913-023-10478-8

**Published:** 2024-01-09

**Authors:** Lindsey de Vos, Joseph Daniels, Avuyonke Gebengu, Laura Mazzola, Birgitta Gleeson, Benjamin Blümel, Jérémie Piton, Mandisa Mdingi, Ranjana M.S. Gigi, Cecilia Ferreyra, Jeffrey D. Klausner, Remco P.H. Peters

**Affiliations:** 1 Research Unit, Foundation for Professional Development, East London, South Africa; 2 Edson College of Nursing and Health Innovation, Arizona State University, Phoenix, United States of America; 3 FIND, Geneva, Switzerland; 4 Institute of Social and Preventive Medicine, University of Bern, Bern, Switzerland; 5 Keck School of Medicine, University of Southern California, Los Angeles, United States of America; 6 Department of Medical Microbiology, University of Pretoria, Pretoria, South Africa; 7 Division of Medical Microbiology, University of Cape Town, Cape Town, South Africa

**Keywords:** *Neisseria gonorrhoeae*, Sexually transmitted infections, Point of care test, Implementation, Qualitative, Normalization process theory, South Africa

## Abstract

**Background:**

South Africa maintains an integrated health system where syndromic management of sexually transmitted infections (STI) is the standard of care. An estimated 2 million cases of *Neisseria gonorrhoeae (N. gonorrhoeae)* occur in South Africa every year. Point-of-care diagnostic tests (POCT) may address existing STI control limitations such as overtreatment and missed cases. Subsequently, a rapid lateral flow assay with fluorescence-based detection (NG-LFA) with a prototype reader was developed for *N. gonorrhoeae* detection showing excellent performance and high usability; however, a better understanding is needed for device implementation and integration into clinics.

**Methods:**

A qualitative, time-series assessment using 66 in-depth interviews was conducted among 25 trained healthcare workers involved in the implementation of the NG-LFA. Findings were informed by the Normalization Process Theory (NPT) as per relevant contextual (strategic intentions, adaptive execution, and negotiation capacity) and procedural constructs (coherence, cognitive participation, collective action, reflexive monitoring) to examine device implementation within primary healthcare levels. Interviews were audio-recorded, transcribed, and then analyzed using a thematic approach guided by NPT to interpret results.

**Results:**

Overall, healthcare workers agreed that STI POCT could guide better STI clinical decision-making, with consideration for clinic integration such as space constraints, patient flow, and workload. Perceived NG-LFA benefits included enhanced patient receptivity and STI knowledge. Further, healthcare workers reflected on the suitability of the NG-LFA given current limitations with integrated primary care. Recommendations included sufficient STI education, and appropriate departments for first points of entry for STI screening.

**Conclusions:**

The collective action and participation by healthcare workers in the implementation of the NG-LFA revealed adaptive execution within the current facility environment including team compositions, facility-staff receptivity, and STI management experiences. User experiences support future clinic service integration, highlighting the importance of further assessing patient-provider communication for STI care, organizational readiness, and identification of relevant departments for STI screening.

**Supplementary Information:**

The online version contains supplementary material available at 10.1186/s12913-023-10478-8.

## Background

Low-and-middle-income countries share the highest burden of sexually transmitted infections (STIs), with *Neisseria gonorrhoeae* (*N. gonorrhoeae*) estimated to be higher in the African region as compared to other regions [1, 2]. Untreated STIs can lead to pelvic inflammatory disease, infertility, and other adverse pregnancy and birth outcomes; also, these may facilitate HIV transmission and acquisition [3–5]. Provider-initiated symptom screening and syndromic management of STIs is currently the standard of care and is widely implemented in resource-constrained settings per WHO guidelines [6–8]. South Africa, and most other resource-constrained countries, has integrated syndromic STI screening for patients at primary care to improve accessibility and efficiency. Although relatively cheap and easy to implement, the syndromic STI approach is associated with unnecessary use of antibiotics, impacting on antimicrobial resistance (AMR), and resulting in asymptomatic infections being untreated [9, 10].

Etiological testing and treatment of infections such as STIs is often challenging in resource-constrained settings due to costs, poor access to laboratory testing, and long turnaround times [3, 11]. Molecular diagnostic tests (e.g. Polymerase chain reaction) are available, but these are often expensive, require a skilled technical workforce and have longer processing and turnaround times [12]. To date, point-of-care STI testing for many low-and-middle-income countries primary care settings is often limited to HIV and syphilis antibody rapid tests. A rapid, affordable, and easy-to-use *N. gonorrhoeae* point-of-care test (POCT) with high sensitivity and specificity could increase access to etiological diagnosis, improve STI treatment appropriateness and outcome, and reduce risks for ongoing transmission and lost-to-follow-up [11–14]. In response to the need for *N. gonorrhoeae* POCT, FIND together with the World Health Organization (WHO) developed a *N. gonorrhoeae* POCT target product profile [15]. Subsequently, a rapid lateral flow assay with fluorescence-based detection (NG-LFA) with a prototype reader was developed by FIND for *N. gonorrhoeae* detection [16, 17]. The NG-LFA showed excellent performance (sensitivity > 90%, specificity > 95%), and test accuracy amongst symptomatic male and female patients visiting South African primary healthcare facilities [18]. Thus far, the NG-LFA has shown high learnability, usability and acceptability amongst trained healthcare professionals and fieldworkers integrated within public healthcare facilities [19, 20].

Importantly, the availability of accurate and sensitive STI POCTs does not guarantee adoption by end-users in routine care. Despite good diagnostic performance, implementation challenges were previously reported amongst antenatal syphilis screening, malaria POCTs, new TB treatment programs and HIV testing and counselling interventions [20–24]. Although STI testing and specimen collection may be feasible and acceptable amongst primary care patients, the required resources and facility infrastructure for test execution, result interpretation, patient flow, and perceived impact pose important considerations for integration and adoption [25–28]. Barriers for implementation may further include issues with leadership, user willingness, consideration for workloads, and privacy challenges [13, 20–22, 29–33]. Exploring healthcare provider perceptions, implementation processes and social and organizational factors will reveal provider beliefs and practices that inform best strategies to ‘normalize’ rapid *N. gonorrhoeae* POCT amongst patients [16, 28, 29, 33, 34].

The following qualitative study unpacks the end-user’s experience of the NG-LFA within primary healthcare facilities and provides critical assessments to inform integration and perceived impact for STI management in South African healthcare settings. Furthermore, specific context and end-user values may inform developers of appropriateness and adaptability as POCTs become more readily available [31, 35].

## Methods

### Study setting and implementing healthcare workers

**Fig. 1:**
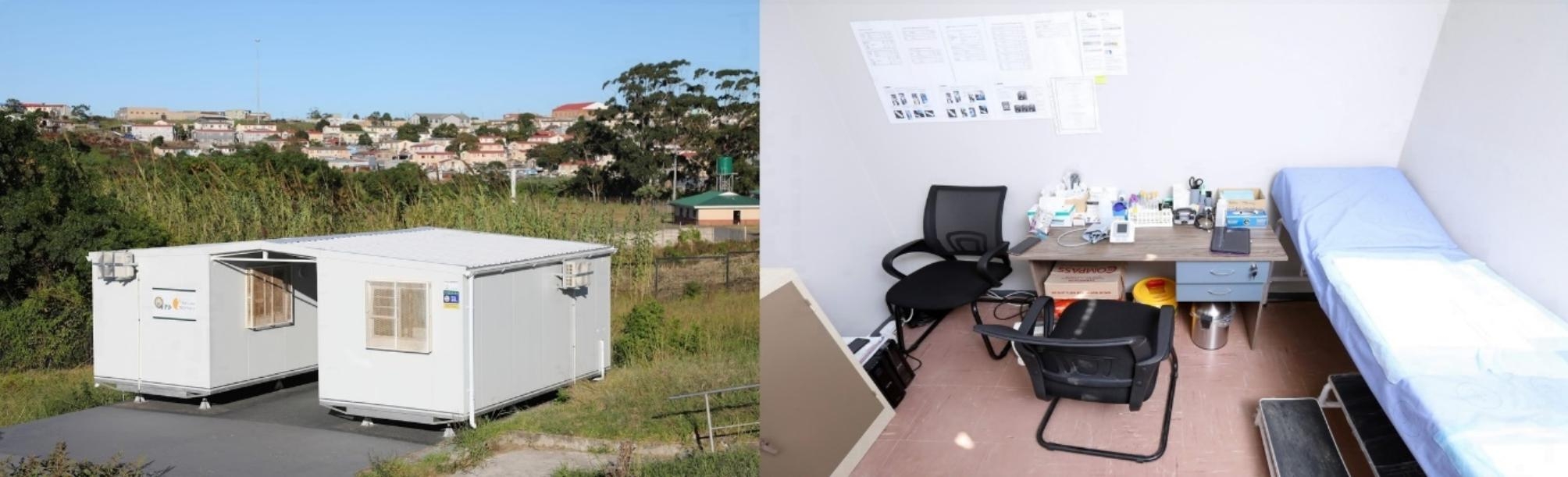
Study setting and site infrastructure for STI screening and testing

A series of in-depth interviews were conducted with healthcare workers directly or indirectly involved in the implementation of the NG-LFA at five primary healthcare facilities in the Eastern Cape, South Africa (Fig. 1). This qualitative component was part of a cross-sectional study assessing the diagnostic performance of the NG-LFA between November 2021 – September 2022. GeneXpert-IV® (Cepheid, Sunnyvale, CA) was used as the gold standard test evaluating the performance of the NG-LFA and healthcare workers were trained on the Xpert prior to the implementation of the NG-LFA.

The study aimed to gather experiences on two distinct healthcare worker levels: healthcare professionals (HCPs; doctors and registered nurses with 10 + years work experience) and field workers (FWs; with prior experience in social or health programs). Healthcare professionals were primarily responsible for primary healthcare services including basic antenatal care, STI screening and treatment, and family planning. The HCPs were assisted with administrative duties and STI testing by FWs. Data collection and analysis was guided by the Normalization Process Theory (NPT) which highlights implementation processes as per end-user experiences [29, 34], that help inform first steps for facility integration.

### The NG-LFA device

**Fig. 2:**
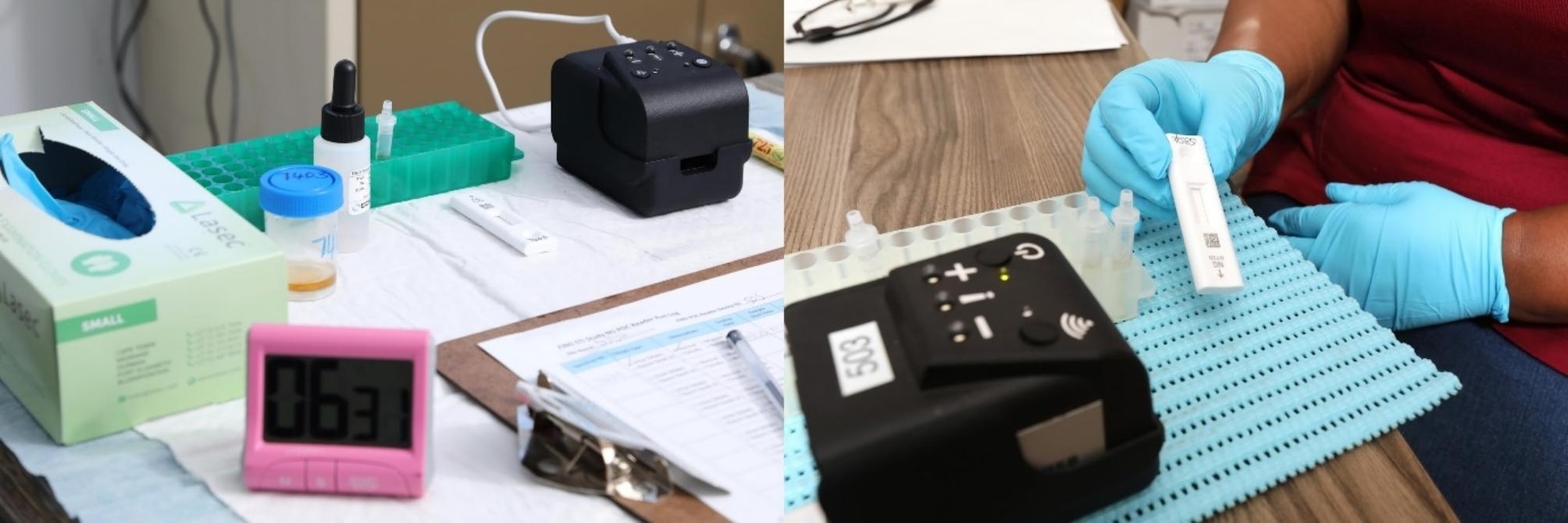
NG-LFA device and testing set-up

The prototype NG-LFA device and reader were developed by FIND to support *N. gonorrhoeae* diagnosis and guide antibiotic use in low-resource settings [15]. The NG-LFA is a single-use disposable test cassette housing a fluorescent lateral flow assay and a portable POC reader for the qualitative detection of *N. gonorrhoeae* (indicating either a positive, negative, or invalid test result) within 20 min [17, 18]. Healthcare workers were given timers, test tube racks, specimen collection kits (rayon swabs for female specimens and urine jars and transfer pipets for male specimens), bottles containing lysis buffer, and tubes with separate dropper caps for testing preparation (Fig. 2). Healthcare workers were provided with control cassettes (positive and negative) and a battery power bank to power the POC reader. After the NG-LFA has run for 20 min, results are automatically available within seconds upon cassette insertion. The testing process and materials have been described in detail previously [19].

### The conceptual framework

NPT was used as an interpretive lens that describes how novel health approaches or technologies are used collectively by individuals and reveals how potential factors either promote or hamper integration into routine care [29, 34]. NPT looks at implementation context, mechanisms (processes), and outcomes using a sociological approach and has been widely applied to novel healthcare interventions including HIV testing, POCT integration, and STI screening [21, 23, 33, 36]. The NPT contextual constructs of strategic intention, adaptive execution, and negotiation capacity informed the prospective facility-specific implementation of the NG-LFA.

**Fig. 3:**
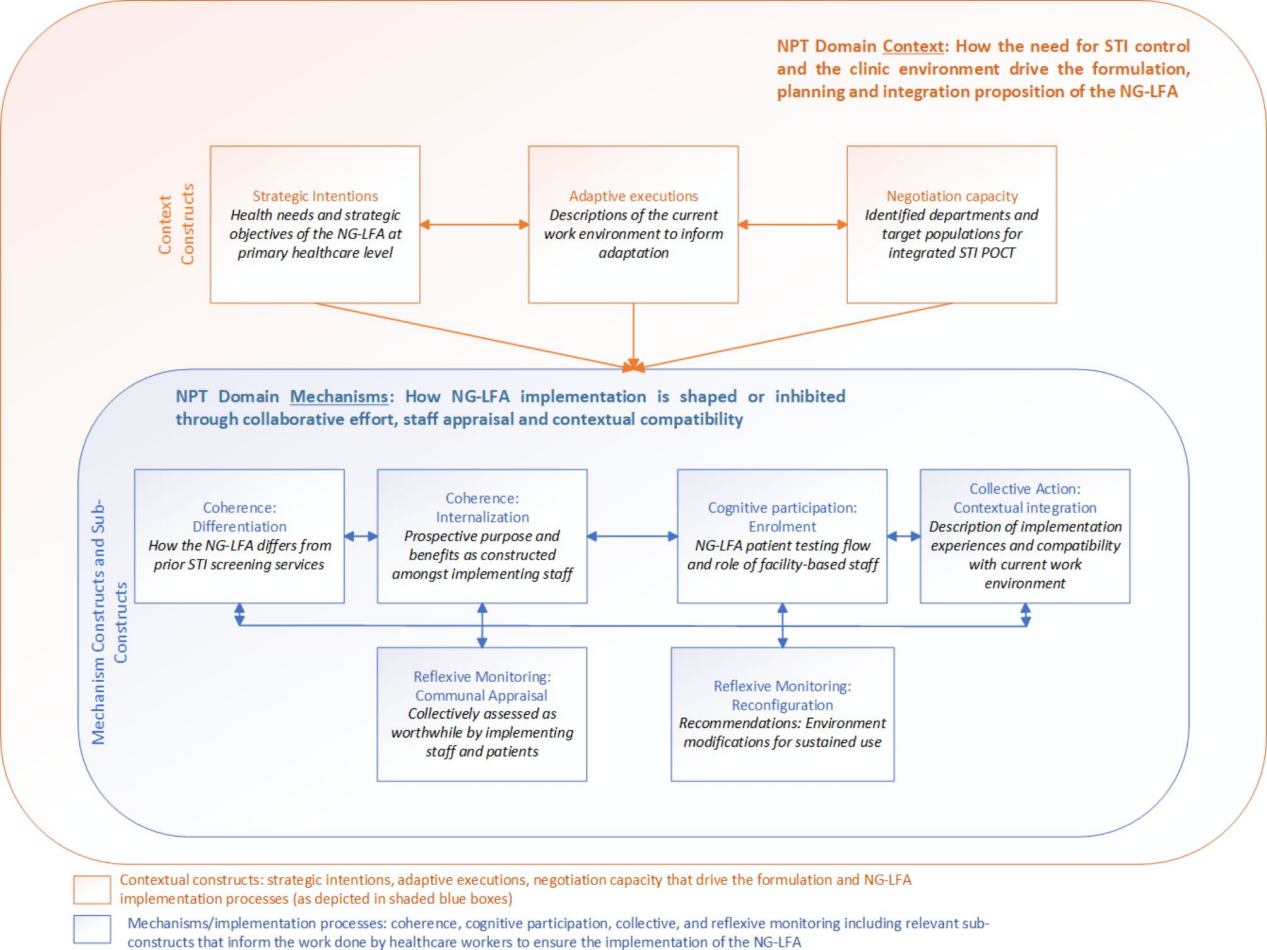
Adapted Normalization Process Theory model: including relevant Contextual and Process (Mechanisms) Constructs. Constructs relevant for understanding the implementation and proposed integration of the NG-LFA at primary healthcare leve

Implementation was informed by provider-patient dynamics, workflow, and public healthcare staff receptivity – including perceived benefits and preferences by implementing healthcare workers. This data was organized by NPT constructs of coherence building, cognitive participation, and collective action as well as reflexive monitoring. For deeper understanding of sub-processes, and implementation dynamics, mechanism sub-constructs were also included during analysis. All constructs and sub-constructs are described in Fig. 3, adapted from the NPT implementation domains and constructs [34].The study is not able to examine outcomes or structural changes at the facility level at this point.

#### Study site capacity (building) training

Both HCPs and FWs were trained to use the NG-LFA by attending several lectures on *N. gonorrhoeae* and the study protocol [19] as well as a three-hour practical group workshop led by study investigators. Quick cards guided testing steps. Healthcare workers were able to practice specimen handling, preparation, and result interpretation with supervision, using control cassettes. Before implementation, on-site preparations, service integration discussions with facility staff and the team, and follow-up training were done. All new employees hired during implementation received a one-day group training on the study protocol followed by shadowing previously trained healthcare workers on-site for at least two days. NG-LFA testing materials were delivered by management staff once patient recruitment was active at a specific facility. The management staff was available for stock orders, troubleshooting, and discussions of clinical case studies.

#### Study site set-up and implementation

Healthcare workers were integrated into the collaborating primary healthcare facilities and community healthcare centers in the Buffalo City Metropolitan Health District, in agreement with facility- and operational managers, adhering to facility protocols during implementation. Patient recruitment, specimen collection, and patient consultations were either conducted in a dedicated study container, study room within the clinic or shared clinic consultation room. Healthcare workers collaborated with clinic-based staff to identify potential eligible adult male and female patients (≥ 18 years) presenting at the clinic with male urethritis syndrome or vaginal discharge syndrome and who were willing to screen for STIs. Both HCPs and FWs had similar implementation responsibilities. They prepared and ran the NG-LFA test for patients as they presented at the facility, with nurse-collected specimens from physical examinations, including vaginal swabs and first-void urine for male patients. NG-LFA test results were confirmed, and treatment was directed by results from the gold standard test only. Patients also received a partner notification slip for partner testing and treatment referral. Any patient that was not willing to wait for their Xpert results were given syndromic treatment as per the standard of care. Test results were documented from the NG-LFA reader and captured on REDCap [37, 38].

### Data collection

#### In-depth interviews

Healthcare workers were purposively sampled and invited to provide in-depth information on NG-LFA implementation at four study time points (post-training, initial use, 3- and 6-month implementation) either in-person, telephonically or via a virtual call. Semi-structured in-depth interview (IDI) protocols (Additional File 1) including probes were developed to assess expectations, processes for planning and implementation, patient dynamics, workflow, clinic-staff receptivity, implications for scale-up, and perceived benefits. Participants were consented using an informed consent form and made aware that they would be asked about their experiences using this novel device among patients. IDIs were conducted in a private location by one of two interviewers not directly involved in implementation and IDIs lasted approximately 25–40 min. All IDIs were conducted in English, transcribed, and reviewed for accuracy. Interview guides and data collection processes were iteratively refined throughout study implementation.

### Data analysis

Initial transcripts were open-coded to develop a codebook. Codes were applied to all transcripts by the qualitative team (JD, LDV, and AG) using Dedoose [Version 9.0.17] [39] and the final codebook was iteratively refined during data collection. The main analytical focus included user experiences, device handling, patient-provider interactions, and clinical considerations. The qualitative data and themes were iteratively refined through team discussions and matrices. The NPT allowed for a more in-depth thematic analysis of the qualitative findings to assess how the NG-LFA was implemented at each site and the factors that highlight preferences, influence use, and potential integration. Available qualitative data helped to understand both the implementation context and mechanisms, and these were organized according to the relevant NPT constructs. Responses between field workers and healthcare professionals were evaluated using a constant comparison approach [40]. Findings and emerging themes were regularly presented to study investigators and iteratively refined through team meetings. Study details are reported as per the Consolidated Criteria for Reporting Qualitative Research (COREQ) guidelines (Additional Table 1) [41].

### Participant in-text representation

Qualitative findings are presented on organizational, provider- and patient-levels. Relevant themes as described here are further aligned with the NPT in the discussion. In-text participant quotes are represented as healthcare worker type (e.g., FW 1, HCP 1) and interview time point (pre-implementation, initial use, or after 3- or 6-month study implementation).

## Results

A total of 66 IDIs were conducted across the time-series usability assessment. Overall, this included 11 HCPs and 14 FWs who were interviewed several times throughout implementation depending on availability and timing of joining the study. No differences were identified in end-user experiences between HCPs and FWs regarding implementation, however, HCPs were able to provide more insight into the impact on clinical management of patients.

### Organizational level

#### Patient flow and teamwork

HCPs and FWs worked in pairs and alongside government facility-based staff to support the integration of the device within primary healthcare facility dedicated spaces. Amidst their clinical duties, HCPs were called to assist with specimen collection and treatment provision:

*“…I would take the participant to the research nurse and then she would take all the swabs, and everything that is needed, the urine and everything, then I would take all the swabs that need to be tested on the lateral flow and in the meantime the nurse would do the urine on the GeneXpert machine.”* [FW 1, Initial use].

This demonstrates FWs capability to run the STI rapid test but had to rely on healthcare professionals for symptom evaluation, specimen collection and corresponding clinical management. To optimize the services provided to patients and ensure STI testing did not increase patient waiting times, the STI tests were run consecutively with other health services:

*“we take… the swabs and then we would send them [the participants] for HIV testing… so by the time they come back our test is ready, and then we give them the results and we give them treatment when necessary.”* [HCP 1, Post-].

Further, several members discussed how the NG-LFA was received by government staff. Healthcare workers initially felt that the NG-LFA would be well received due to recurring symptomatic cases. Particularly because intensive screening and physical examinations may not be done routinely for STIs:

*“…the only thing that will be done at the clinic, they will just give them the syndromic approach and maybe explain the hygiene or what she must do but they will never explain that I’m suspecting that you’ve got this and this and it is caused by this, and your treatment will be this…”* [HCP 2, Initial-].

*“…some have taken it very well because they refer us patients that… they have seen that they have been treating this participant maybe for quite some time but there is no improvement, so they refer them to us ‘now that you have the machine can you test this participant and see what… really [is] happening.”* [HCP 3, Mid].

Targeted STI screening was perceived as particularly beneficial for the healthcare facility, and when facility staff are unable to offer patients relief using the syndromic approach.

### End-users/ staff value

#### Organizational composition and specimen collection

The public healthcare system in South Africa is largely under-resourced which affects service delivery, waiting times, and private space for patients. Some healthcare workers felt that to make a device such as the NG-LFA a workable proposition; additional private space is required for the collection of specimens and administering STI treatment.

*“[for] the treatment of the gonorrhea we [will] need private places for people to give us samples and specimens… as well as counseling facilities to include their partners in the treatment or to come here.”* [HCP 4, Pre-].

*“…But with the issue of not having an actual room to work or space some [clinic staff] are still reluctant to refer [patients] or come to say, “can you check this one because this guy is having a problem…””* [HCP 3, Mid].

Dedicated spaces may optimize the reach of the NG-LFA test for specific departments, yet STI screening also requires additional resources for partner counseling and treatment.

Although there were initial space constraint concerns and queries for service integration, the NG-LFA was able to suit the space provided. As the NG-LFA was perceived as a small and portable device, healthcare workers did not recommend structural changes and adapted to the current work conditions:

*“It’s very suitable because we put our container [the LFA storage box] on a cupboard… we just take our container with us, go where, maybe the participant is on the youth side it’s easy for us to take our container and go there”* [FW 2, Post-].

Healthcare workers were probed about the prospects of nurse- versus self-collected specimens for NG-LFA integration. Given prior experience with Xpert specimen collection and ease of urine collection, healthcare professionals felt that specimens for NG-LFA testing would be well-received and not perceived as invasive. Although self-collected specimens might be suitable given space constraints and testing hesitancy, such as male patients who show reluctance for physical examinations and swabs required for additional clinical assessments, “*…I think they [men] would prefer the urine part not the taking of swab… but when taking the swabs, the nurse has to do that”*, [FW 1, Post-]. The majority of healthcare workers felt nurse-collected swabs was ultimately more trusted:

*“…Self-collected uh it’s mainly judged by pain [for female patients] because… if they start feeling the pain they retract and then they say they’re done….that is when we will get these um results that are not quite like with the symptoms that are suggestive of NG, but you get results that are negative then you’re like what is going on?”* [HCP 5, Post-].

*“…of course, I trust the nurses more… because they know exactly how far the thing… [vaginal swab] should go in but… I didn’t hear them complain of any issues like maybe there’s not enough specimen there on the swab.”* [FW 3, Post-].

There was a perception that nurses were able to obtain sufficient ‘sample’ collecting vaginal swabs for valid test results than if patients were to collect swab specimens themselves.

#### Perceived implications for clinical care

Several healthcare workers felt the availability of a rapid STI POCT at the primary healthcare level could increase STI detection and address to some extent the issue of reoccurrence by being able to test for specific STIs. Those who practiced using the NG-LFA reiterated the need for efficient STI screening and antibiotic use given the high burden of STIs in the study area:

*“I think a physical and practical way of finding, detecting gonorrhea in patients with symptoms and also to minimize the recurrence of it because with the syndromic approach… you find that a person comes over again and again and again, due to lack of information of what they really are suffering from because now we just take it as an STI, we are not even sure which STI it is…”* [HCP 5, Pre-].

Over time, the NG-LFA was perceived to have the potential to assist nurses in more accurate diagnostic and treatment provision, where POCT would further contribute to better clinical management of symptomatic and asymptomatic patients, whereby one *“know[s] what you are treating rather than beating around the bush not knowing”* [HCP 3, Mid-]; and that it’s *“more helpful to provide clear guidance on what treatment to use.”* [HCP 6, Mid].

### Perceived patient benefits

#### Increased STI awareness and reduced testing apprehension

STI POCT was perceived to increase STI knowledge amongst patients as compared to the standard of care/syndromic approach. Patients were often described as not being very knowledgeable of different STIs and were only made aware of relevant symptoms (e.g., discharge) they had:

*“They know drop, any male they only know drop. They know there’s different types of drop… you get drop, it can be STI it can be chlamydia, it can be NG it can be something else, no they only know that it’s drop. With the females its infection, they don’t know if it’s chlamydia if it’s TV [trichomoniasis]…”* [HCP 5, Post-].

Similarly, POCT may increase the awareness of different STIs for healthcare professionals more familiar with the guidelines for syndromic management:

*“So now I started with the team for STIs, I wanted to know, I didn’t know there was something called chlamydia, I only knew there are STIs, I only knew VDS [Vaginal Discharge Syndrome], MUS [Male Urethritis Syndrome], I didn’t know there was chlamydia, gonorrhea… and there are different managements [of STIs].”* [HCP 3, Pre-].

Healthcare workers provided patients with targeted treatment as per the South African guidelines depending on the type of infections. Furthermore, STI POCT could address STI testing apprehension and potential concerns about stigma due to its ability to test quickly and without drawing attention, as if offered as part of routine care. Several healthcare workers felt the test would make patients less shy to test and more keen to resolve their symptoms:

*“I think if they [the LFA] were used regularly at the clinic they would, they [patients] were also not going to be shy you know to come, because most of them they are shy, they only come when there is really a problem.”* [HCP 2, Initial].

*“I think it changes them because they are not ashamed anymore because back then you would find that if you said you have STI then they are so closed they don’t want to talk to anyone about it, they just want it to be over… but now they are so open about it, “okay I want to test, what are you doing.””* [FW 4, Initial].

Frequent, easily accessible, and rapid testing integrated in routine services, accompanied by more STI information, was perceived to have the potential to ‘normalize’ STI-targeted screening for patients within facilities.

#### Device receptivity and familiarity with other rapid tests

The largest benefit of STI rapid testing for healthcare workers was the provision of quick results and the patient’s willingness to wait for the results:

*“…people don’t usually wait for their results, so you find out that if you are calling the participant to come to the hospital, she will take about a month or two to come… So with this new system, its easy, you test the person and you get positive results and then will you treat the person immediately.”* [FW 5, Pre-].

*“…the time, that’s the biggest advantage and the fact that using this is like bringing something that was not already there, like STI testing other than HIV and syphilis that’s something that has never been done in this part of the world so it’s something that will especially be available to the public better, it will be very beneficial to the community…”* [HCP 5, Mid].

They described the implications of long waiting times for standard laboratory or PCR results that delay patient presentation for treating STI symptoms. A rapid test for STIs was thus perceived to yield higher testing and treatment receptivity by being more accessible and being able to provide same-day screening and treatment.

Throughout implementation, healthcare workers gave examples of comparing the NG-LFA to HIV, syphilis, and pregnancy rapid testing, as well as Xpert STI POC. Staff felt that the NG-LFA was a device that patients could easily understand and identify. Healthcare workers discussed perceived value in showing the device and testing process to patients:

*“…unlike the GeneXpert they don’t know what’s going on, at least now they will see that we took the swabs and then put it in the device right in front of their eyes, so I think they might trust it more…”* [FW 3, Pre-].

*“So I explain to them that this is a new test so that we can get STI results very quickly, I compare it to HIV test because that’s something they are all familiar with, I mean the time frames are similar, the HIV test is 15 minutes and this one is 20 minutes…”* [HCP 7, Initial].

Healthcare workers felt that testing and specimen collection transparency, and familiarity with other rapid tests would ensure trust in the NG-LFA amongst patients, even when the test was still new and undergoing performance testing.

### Recommendations for next steps

#### POCT service integration

After 6 months of implementation, healthcare workers were asked to reflect on STI screening as part of existing routine services. When prompted, they also felt that STI testing could be integrated with HIV counselling and testing, as this is often a first entry point for patients at the primary healthcare level covering several departments and target populations. Healthcare workers gave examples of several departments for device integration including observations, family planning or integrated as part of routine care as offered by professional nurses at primary healthcare level:

*“I think that facility would treat as many people as possible because if for instance the scenario we were talking about testing everyone coming for HIV test that would mean that uh the clinic would get more people and people would be helped (for STIs).”* [FW 6, Post-].

*“They are the initial point of care [observations] for these patients and with those that need to start at the nurses offices… so to save time it would be best when a patient is complaining of such [symptoms], they are referred to those people at observation to conduct this test, the swabs are given to them, like the nurse would collect the swabs themselves…”* [HCP 5, Post-].

*“…they have two nurses for the youth department, they deal with family planning and pap smears so I think it would be more convenient for them.”* [HCP 8, Post-].

The incorporation of STI rapid testing was perceived to be particularly beneficial in youth-friendly services as many patients presenting with STI-related symptoms were referred from these departments. These also included family planning, for the perceived need and opportunity to collect specimens during physical examinations (e.g., in addition to pap smears).

#### Dedicated departments versus integrated care for STI screening

However, an important consideration as suggested by a few healthcare professionals was the identification of appointed healthcare providers for rapid STI screening and care. This also included potential implications on holistic care as well as the time spent with patients:

*“…we do one thing in one room we will be treating our patients holistically… then we’d be integrating even the STI because you’ll find out that in the facility they would say, “no there will be a specific room that will be testing STI”… and you were not there when they were testing that participant, you just take the result as it is because you’re not the actual person who was doing the test, that’s where we need to change the health system.”* [HCP 3, Post-].

*“…what is happening in the clinics each and every nurse is attending to all the clients like the acute, the chronic and the adolescents and youth if a person comes to your consulting room you do everything, neh?[Right?]*. *So I think then maybe really they will complain of time… unless it could be dedicated to, as I’ve said the stream of adolescence and youth you know.”* [HCP 2, Post-].

Healthcare workers highlighted the potential pros and cons between integrated STI screening in routine services and/or having dedicated departments cater to STIs. This further shows the importance of assessing end-user receptivity and the allocation of clear responsibilities for successful integration of a novel test device.

**Fig. 4:**
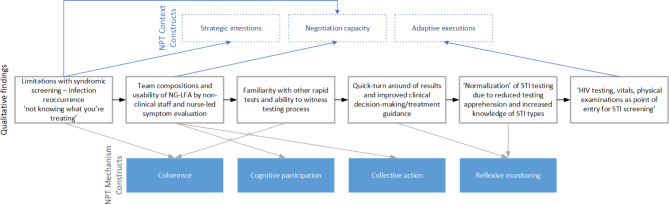
Summary of qualitative findings as per Normalization Process Theory constructs. Description of implementation considerations for sexually transmitted infection point-of-care screening and implications for clinical management

## Discussion

This qualitative study is the first to describe the implementation experiences of a novel *N. gonorrhoeae* rapid POCT within the South African healthcare environment. Implementation and end-user receptivity amongst trained healthcare workers was evaluated through varying organizational levels using the specific features of this NG-LFA and the general features of the target product profile [15]. Rather than proposing structural change, the findings show adaptive execution within the current clinic environment and health programs.

Figure 4 and Additional Table 2 describes how the facility environment and implementation experiences inform the NPT constructs and the proposed process of moving from syndromic to targeted treatment and care for STIs in primary healthcare. Implementing healthcare workers described the *strategic intentions* for the NG-LFA during pre-implementation and initial use by reiterating the need for STI POCT given limitations with syndromic management [11]. This was further shared by examples from government clinic-staff of patients who referred patients with recurring symptoms, whereby the NG-LFA could offer (immediate) resolution [13]. Study healthcare workers relied on the existing patient flow and worked alongside government clinic staff to successfully implement the NG-LFA within the existing health structure. This demonstrates preliminary *negotiating capacities* or buy-in, as well as the *collective execution and participation* of the *N. gonorrhoeae* test. The study healthcare professionals led the diagnostic test evaluation with the assistance of the fieldworkers that optimized the use of the test [42]. Further investigations for evaluating the performance of the NG-LFA and patient preferences may be required to assess the possibility for asymptomatic testing services and self-collected specimens given space constraints, and for task-shifting opportunities from professional nurses to paraprofessionals. However, these must not compromise end-user and patient trust of the device [35].

This study raises the importance of assessing organizational readiness, designated responsibilities, and service flow that ensures STI screening and preventative care is given adequate attention [20]. Given the lack of STI targeted testing and screening at primary healthcare, we begin to address this by showing how the NG-LFA best fits within the current context. Aligning the NG-LFA test with existing and relevant patient services such as HIV counselling and testing, checking for vitals, and family planning could for instance help streamline STI screening specimen collection and long patient waiting times. Paraprofessionals involved in these services could test for STIs as similarly performed for HIV where self-collected specimens would remove the dependence on professional nurses to execute testing. Healthcare services such as physical examinations during antenatal care of family planning (e.g., pap smears), would also provide a conducive opportunity for vaginal specimen collection. Further, the NG-LFA was perceived as suitable given resource limitations and the ability to refer patients for other services whilst POCTs are executed [30].

However, when considering the *adaptive execution* of the NG-LFA (and how the environment informs this adaptation), some healthcare workers discussed the impact of adding another testing service on a burdened healthcare system sustained by professional nurses. In South Africa, integrated care ensures that patients largely receive efficient and holistic care from the same provider. However, some concerns were raised for increased workloads [43]. Some health care professionals felt the NG-LFA would be more appropriate for dedicated departments (vertical implementation) due to convenience and perceived need (e.g., youth and adolescent services, family planning etc.) [44]. Others mentioned it was important to offer comprehensive care or clinical oversight for a patient for STI care and thus felt STI screening should be part of integrated care. This shows that the existing context may influence how each clinic may establish different workarounds for how the NG-LFA could best be integrated [28].

Implementing healthcare workers were able to draw comparisons between Xpert and other rapid tests (e.g., for HIV), particularly highlighting some differences for current specimen collection procedures, quick results, and the easy interpretation of the results for the NG-LFA. These differences and perceived benefits for the NG-LFA depicted healthcare workers’ *coherence and internalization* of POCT for *N. gonorrhoeae*. As shown in previous POCT studies, awareness and observability of the NG-LFA testing procedures appeared to promote patient and provider satisfaction and trust in the device [32, 36, 45]. As intended with proposed target product profile, the NG-LFA was also perceived to guide better clinical management. Similarly to experiences amongst other healthcare workers using POCTs in Uganda, staff appreciated the clarity for treatment provided by the NG-LFA and described more confidence and efficacy in their clinical decision-making [13, 46].

During the *reflexive monitoring process*, both healthcare professionals and field workers shared the perception that an easily accessible STI POCT at the primary healthcare level could allay testing apprehension [32], also caused by long waiting times for test results [30]. Feedback indicates that testing receptivity is increased with STI testing that is clearly described, quick, inconspicuous, accurate, and which enables fast diagnosis for those presenting with symptoms – in addition to reducing stigma and improving same-day treatment [13, 42, 47, 48]. Further, healthcare workers described how diagnostic testing for *N. gonorrhoeae* would create STI awareness amongst providers and patients as previously found with molecular tests [13], and highlights the importance of conducting physical examinations during primary care. The implementation of STI POCTs however begs consideration for additional resources for targeted treatment protocols and partner referral procedures, such as designated private spaces for STI treatment, partner notification, and testing.

### Strengths and limitations

This qualitative study included a large sample of interviews conducted at different timepoints that offered in-depth implementation experiences using the NG-LFA over a six-month timeframe. Findings from this study reveal key social and organizational interactions at primary health-care levels with implications for current STI testing and treatment guidelines. The implementation context is unique to the South African primary health care system but may provide insights for similar low- and middle-income health systems.

The current study design does not allow for the assessment of the intervention performance, restructuring and sustainment of the NG-LFA within primary healthcare. Not addressed here are important post-implementation issues relating to stock-outs and quality assurance for testing in context of routine service delivery, as these were directly controlled by study staff. While this study marks the initial step to delineate end-user encounters of a novel rapid POCT in a primary healthcare setting, it is important to explore the preferences and experiences of patients as well as key informants during device development. These could further enhance our understanding of patient and provider acceptance, partner testing, and clinical management. Additionally, organizational readiness and the cost-effectiveness of targeted STI treatment within primary care warrants further investigation [21, 22, 35].

## Conclusion

Before a novel POCT device can be integrated into primary care, it is critical to understand user acceptability and implementation experiences to complement device performance and accuracy. The NPT framework enabled a more detailed understanding of the context and implementation processes of the NG-LFA, as well as potential adoption of the device by both professional nurses and non-clinically trained fieldworkers. The NPT analysis reveals critical gaps, requirements, and next steps for potential scale-up of an STI POCT into organizational structures for integrated versus specialized care.

### Electronic supplementary material

Below is the link to the electronic supplementary material.

**Additional File 1**: IDI protocols. Interview protocols used in this study during the pre-implementation, initial use, 3-month (mid-assessment) and 6-month (post-evaluation) implementation phase

**Additional Table 1**: Consolidated criteria for reporting qualitative studies (COREQ): 32-item checklist for interviews and focus groups

**Additional Table 2**: NPT Constructs as defined for the implementation of the NG-LFA

## Data Availability

The datasets generated and/or analysed during the current study are not publicly available because of the confidential nature of the interview responses and transcripts that may identify a participant. For studies conducted in South Africa, the POPI act poses restrictions on the data that can be shared publicly. Data are available from the corresponding author on reasonable request.

## Abbreviations

AMR: Antimicrobial resistance
BCM-HD: Buffalo City Metropolitan Health District
FPD: Foundation for Professional Development
FWs: Fieldworkers
HCPs: Healthcare professionals
HCWs: Healthcare workers
IDIs: In-depth interviews
NG-LFA: *Neisseria gonorrhoeae* lateral flow assay (and reader)
NPT: Normalization Process Theory
POCT: Point-of-Care Test
STIs: Sexually transmitted infections
WHO: World Health Organization
