# Supplementary material for: Implementation considerations for a point-of-care Neisseria gonorrhoeae rapid diagnostic test at primary healthcare level in South Africa: a qualitative study
Source: BMC Health Serv Res. 2024 Jan 9;24:43. doi: 10.1186/s12913-023-10478-8 (PMC10777514; doi:10.1186/s12913-023-10478-8)
Supplement: Supplementary file 1 — Additional File 1: IDI protocols. Interview protocols used in this study during the pre-implementation, initial use, 3-month (mid-assessment) and 6-month (post-evaluation) implementation phase [file 12913_2023_10478_MOESM1_ESM.pdf]

B. SITE PREP IDIs

Key Informant IDI Protocol – Pre-Implementation

1. Can you please describe your study role? [Introductory]
2. Can you please describe your study facility?
   a. What is the flow and referral processes of patients presenting with STI symptoms?

3. Please describe your level of experience with STI point-of-care testing?
   a. Have you handled any similar devices, please explain?

4. What were your first impressions of the lateral flow assay device for detection of Neisseria gonorrhoeae?

5. Could you please describe in your own words, what the objective is of this device?
   a. What are your expectations of this device?

6. Please describe how preparing the specimens was for you?
   a. Probe around any challenges / previous experience with specimen collection

7. How do you feel about the materials/equipment that was offered to you to be able to conduct the testing?
   a. Did you feel anything was missing?
   b. How did you feel about using the battery pack?
      i. Probe: around electricity and batteries
   c. Was it easy or difficult to start the testing process, and why?

8. What were your impressions of the reader that provides the results?
   a. Please describe how you felt about handling the device?
      i. What did you find easy / what did you find challenging, and why?
   b. Please describe the process of reading the results, did you experience any challenges?
   c. Please describe your level of trust in the device and the test results

9. Please describe the level of information that you felt you needed to be able to operate this device?
   a. What other information do you still feel you need?

10. When do you think technical support should be offered and why?
    E.g. probe around duration and type of support

11. How will this device have an impact on gonorrhea testing and gonorrhea clinical management, compared to what you did before?

12. Please explain how the device will be integrated into your study site?
    a. What preparations are needed?
    b. Please describe the role of each staff member in conducting the test
    c. How will the device impact your work?

13. How do you think patients will feel about this device?

14. Do you have any other suggestions for the device?
    a. What changes would you make to the device, and why?
    b. If no, why are you happy with the current set-up?

15. Do you have any other comments or thoughts that you would like to share with us regarding the training or initial expectations of this device?

Interview Protocol – Post-Testing/ Initial Use

1. Please describe what you found interesting about the gonorrhoea LFA test?
2. Please describe any additional training that you received to conduct the LFA test since the first training in Ndevana?

3. What is the process of identifying potential participants for this study?
    a. How did this work out?
    b. Did you have to modify the flow at any point? Please describe.
    c. *Probe: around recruiting men/women/ operationalize steps*

4. **[Research Nurses]** Please describe your experiences collecting swabs/urine specimens from participants (probe: male and female participants)
    a. Was it easy or difficult to obtain the specimens?
    b. How does this compare to the specimens that are routinely collected for patients in public clinics?
    c. What types of questions did participants ask?
        i. Why do you think they asked these questions?
    d. Did participants show any concerns?

5. Please describe your interactions with the (senior) field workers?

6. Please describe your role in clinically managing participants for STIs?
    a. Did participants have any questions about the final test results?

7. Did you trust the LFA test results?

8. Do you think patient's perceptions have changed about STI testing?
    a. How do you think it will influence how patients trust providers?
    b. Would future patients trust the use of these devices if used in clinics regularly?

9. At this point, do you have any suggestions for the design of the LFA test?

10. Is there anything else that you'd like to share regarding your initial experiences in this study?

Interview Protocol – Mid-assessment

1. Please describe your role in conducting the gonorrhoea rapid test

2. Please describe the step-by-step process of running the gonorrhoea test?
   a. How many tests have you conducted?
   b. How did you feel about conducting these tests?

3. How have you managed the flow of participants?

4. Please describe your experiences with male/female patients

5. What perceptions do patients have of the study site?
   a. Probe around any concerns or motivators for coming to test

6. How was this new device received by clinic staff?
   a. Please describe their role throughout the testing process

7. What are the advantages of using this gonorrhoea lateral flow assay device?
   a. What are the disadvantages?

8. If you think of the current features of the uReader and LFA system
   a. What are your thoughts of the current system now?
   b. How would you rate the durability of the system in your study site

9. Did you experience any challenges or technical problems preforming the tests?
   a. When did you receive technical support?
   b. Did you receive any additional training?

10. What are your current preferences for STI screening?
    a. In your opinion, how accurate is the gonorrhoea lateral flow assay device?

11. How could you have a better experience using the uReader and LFA system?
    a. Based on your testing experiences, do you have any additional recommendations or comments?

**Interview Protocol – Post-evaluation**
1. Was there something that you would’ve liked to have known about before starting this project?<br>a. How do your experiences compare to your initial expectations of this project?<br><br>2. Are there any new staff members that you trained on preforming the NG LFA test, please describe this process? **[might not be applicable to all]**<br><br>3. If you think of the past 6 months, how did you experience the handling and preparation of the specimens for the NG LFA test?<br>a. How does it compare to your initial experiences preparing the test?<br>b. Could you please compare male and female patient sample collection and testing?<br>c. On average, how long was a patient’s participation in this project (incl. relevant clinical assessments and necessary preparations)<br><br>4. Please describe how you powered the NG LFA for testing?<br>a. How many times did you have to charge the battery the past 6 months?<br>b. Would you keep the battery pack as part of the device? Why/why not?<br><br>5. How did you experience reading/interpreting the results on the reader?<br>a. Please describe a time where you experienced an error/invalid?<br>b. Were you ever unsure of the results? Please provide an example.<br>c. Do you have any preferences for the markings on the device that indicate whether the result is a positive, negative or invalid?<br><br>6. How satisfied are you with the NG LFA test?<br>a. What works well/ what does not work well?<br>b. Did any features of the device wear down over 6 months? Were any components replaced? If so please elaborate<br>c. In your opinion, what is the lifespan of the current device?<br>d. Do you have any preferences for the packaging overall?<br>e. If there would be one thing you could change about the test, what would that be?<br>f. How could this NG LFA system be improved? (and/or) What features would be desirable for a future system?<br><br>7. If you compare to Xpert testing, what are the differences and pros and cons of the LFA? And compared to HIV rapid testing?<br><br>8. How would you rate the suitability of the NG LFA in your work environment?<br>a. **Probe: space, security aspects, frequency use, packaged items, containers**<br>b. Would you be interested in continuing to use this device in your work?

c. Please describe the interest level amongst facility-based staff

9. Do you think this test could be implemented in routine care? If so:
   a. For which patient group(s)
   b. Who should operate it
   c. What barriers and opportunities do you foresee? What resources are available, what is missing?
   d. What else would be needed for integration into public healthcare facilities?
   e. How would you envision the flow of patients to receive a NG LFA test?
   f. How would you feel about the following scenario, everyone that receives an HIV test also receives a STI test?
   g. What would be the impact of a having a diagnostic test for STIs?

10. What would patients prefer regarding STI screening and testing? Males/females
    a. **Probe: Obtaining specimens, types of tests, provider type/communication**
    b. What are the implications for self-collected versus nurse-collected specimens?
    c. Based on your experiences, please describe how patients have received information relating to STIs?

11. Could you come up with a good name for the novel test?

12. In your opinion, what are the next steps for the NG LFA test?
