# Supplementary material for: Implementation considerations for a point-of-care Neisseria gonorrhoeae rapid diagnostic test at primary healthcare level in South Africa: a qualitative study
Source: BMC Health Serv Res. 2024 Jan 9;24:43. doi: 10.1186/s12913-023-10478-8 (PMC10777514; doi:10.1186/s12913-023-10478-8)
Supplement: Supplementary file 2 — Additional Table 1: Consolidated criteria for reporting qualitative studies (COREQ): 32-item checklist for interviews and focus groups [file 12913_2023_10478_MOESM2_ESM.docx]

**Additional Table 1. Consolidated criteria for reporting qualitative studies (COREQ): 32-item checklist for interviews and focus groups**

Developed from: Tong A, Sainsbury P, Craig J. Consolidated criteria for reporting qualitative research (COREQ): a 32-item checklist for interviews and focus groups. International Journal for Quality in Health Care. 2007. Volume 19, Number 6: pp. 349 – 357

| No | Item | Guide questions/description | Reported on  Page no. |
| --- | --- | --- | --- |
| Domain 1: Research team and reflexivity | | | |
| Personal Characteristics | | | |
|  | Interviewer/  facilitator | Which author/s conducted the interview or focus group? | **Methods, pg. 9**  “…by one of two interviewers not directly involved in implementation.” Authors: LDV, AG |
|  | Credentials | What were the researcher’s credentials? E.g., PhD, MD | LDV: BA, MSc  AG: BSW  JD: PhD |
|  | Occupation | What was their occupation at the time of the study? | LDV: Qualitative Research Manager  AG: Qualitative Research Assistant  JD: Associate Professor, Qualitative lead |
|  | Gender | Was the researcher male or female? | 2 female/ 1 male |
|  | Experience and training | What experience or training did the researcher have? | Both interviewers were trained in qualitative interviewing skills and analysis with experience in public health research studies. |
| Relationship with participants | | | |
|  | Relationship established | Was a relationship established prior to study commencement? | **Methods, pg. 9**  Interviewers were not directly involved in the implementation of the parent study. |
|  | Participant knowledge of the interviewer | What did the participants know about the researcher? E.g., Personal goals, reasons for doing the research | **Methods, pg. 9**  “Participants were consented using an informed consent form and made aware that they would be asked about their experiences using this novel device among patients.” |
|  | Interviewer characteristics | What characteristics were reported about the interviewer/ facilitator? E.g., Bias, assumptions, reasons, and interests in the research topic | Facilitators were introduced to the interviewees. Interviewers were from the same organization as the implementing staff. No bias, assumptions, or reasons aside from study objectives were reported to participants. |
| Domain 2: Study Design | | | |
| Theoretical framework | | | |
|  | Methodological orientation and Theory | What methodological orientation was stated to underpin the study? e.g., grounded theory, discourse analysis, ethnography, phenomenology, content analysis | Data was collected as a time-series usability assessment, using a constant-comparison approach and findings were iteratively refined. |
| Participant selection | | | |
|  | Sampling | How were participants selected? e.g., purposive, convenience, consecutive, snowball | **Methods, pg. 9**  “Healthcare workers were purposively sampled and invited to provide in-depth information on NG-LFA implementation at four study time points”. |
|  | Method of approach | How were participants approached? e.g., face-to-face, telephone, mail, email | **Methods, pg. 9**  “…either in-person, telephonically or via a virtual call”. |
|  | Sample size | How many participants were in the study? | **Results, pg. 10**  “…11 HCPs and 14 FWs who were interviewed several times throughout implementation depending on availability and timing of joining the study”. |
|  | Non-participation | How many people refused to participate or dropped out? Reasons? | None of the healthcare workers refused to participate. However, not all participants participated in each time-series implementation phase as some transitioned to other projects or joined the project later to fulfill 6 months of implementation. |
| Setting | | | |
|  | Setting of data collection | Where was the data collected? E.g., home, clinic, workplace | Either in a private space at the study facility or via telephone/virtual call. |
|  | Presence of nonparticipants | Was anyone else present besides the participants and researchers? | N/A, IDIs were conducted one-on-one. |
|  | Description of sample | What are the important characteristics of the sample? e.g., demographic data, date | **Methods, pg. 6**  “…The study aimed to gather experiences on two distinct healthcare worker levels: healthcare professionals (HCPs; doctors and registered nurses with 10+ years work experience) and field workers (FWs; with prior experience in social or health programs). Healthcare professionals were primarily responsible for primary healthcare services including basic antenatal care, STI screening and treatment, and family planning. The HCPs were assisted with administrative duties and STI testing by FWs.” |
| Data collection | | | |
|  | Interview guide | Were questions, prompts, guides provided by the authors? Was it pilot tested? | N/A  Interview guides and data collection activities were iteratively refined. |
|  | Repeat interviews | Were repeat interviews carried out? If yes, how many? | **Methods, pg. 9**  “Healthcare workers were purposively sampled and invited to provide in-depth information on NG-LFA implementation at four study time points (post-training, initial use, 3- and 6-month implementation)”. |
|  | Audio/visual recording | Did the research use audio or visual recording to collect the data? | **Declarations, pg. 23**  Data was audio-recorded. “Participants were made aware that each interview would be recorded voluntarily”. |
|  | Field notes | Were field notes made during and/or after the interview or focus group? | N/A |
|  | Duration | What was the duration of the interviews or focus group? | **Methods, pg. 9**  “…IDIs lasted approximately 25-40 minutes”. |
|  | Data saturation | Was data saturation discussed? | Data was collected at four study time points to ensure expectations, processes for planning and implementation were adequately captured, including any change in device perceptions/usability over time. |
|  | Transcripts returned | Were transcripts returned to participants for comment and/or correction? | Transcripts were not returned to participants. However, transcripts were reviewed. |
| Domain 3: analysis and findings | | | |
| Data analysis | | | |
|  | Number of data coders | How many data coders coded the data? | Three coders (LDV, AG and JD) **Methods, pg. 9**  “Transcripts were independently open-coded by three members of the qualitative research team, and codes were discussed, refined and merged into a final codebook, which was applied to all transcripts.” |
|  | Description of the coding tree | Did authors provide a description of the coding tree? | **Methods, pg. 9**  The following describes the coding and analysis steps: “Initial transcripts were open-coded to develop a codebook. Codes were applied to all transcripts by the qualitative team (JD, LDV, and AG) using Dedoose [Version 9.0.17] [39] and the final codebook was iteratively refined during data collection. The main analytical focus included user experiences, device handling, patient-provider interactions, and clinical considerations.” |
|  | Derivation of themes | Were themes identified in advance or derived from the data? | **Methods pg. 9-10**  “The qualitative data and themes were iteratively refined through team discussions and matrices. The NPT allowed for a more in-depth thematic analysis of the qualitative findings to assess how the NG-LFA was implemented at each site and the factors that highlight preferences, influence use, and potential integration.” |
|  | Software | What software, if applicable, was used to manage the data? | Codebooks were developed and applied to transcripts using Dedoose. |
|  | Participant checking | Did participants provide feedback on the findings? | N/A, however preliminary findings and prior publications were shared with staff. |
| Reporting | | | |
|  | Quotations presented | Were participant quotations presented to illustrate the themes / findings? Was each quotation identified? E.g. Participant number | **Results pg. 10**  “In-text participant quotes are represented as healthcare worker type (e.g., FW 1, HCP 1) and interview time point (pre-implementation, initial use, or after 3- or 6-month study implementation).” |
|  | Data and findings consistent | Was there consistency between the data presented and the findings? | Yes  **Methods pg. 10**  “Available qualitative data informed both the implementation context and mechanisms, and these were organized according to the relevant NPT constructs.” |
|  | Clarity of major themes | Were major themes clearly presented in the findings? | **Methods pg. 10**  “Qualitative findings are presented on organizational, provider- and patient-levels. Relevant themes as described here are further aligned with the NPT in the discussion.” |
|  | Clarity of minor themes | Is there a description of diverse cases or discussion of minor themes? | N/A |
