# Supplementary material for: Implementation considerations for a point-of-care Neisseria gonorrhoeae rapid diagnostic test at primary healthcare level in South Africa: a qualitative study
Source: BMC Health Serv Res. 2024 Jan 9;24:43. doi: 10.1186/s12913-023-10478-8 (PMC10777514; doi:10.1186/s12913-023-10478-8)
Supplement: Supplementary file 3 — Additional Table 2: NPT Constructs as defined for the implementation of the NG-LFA [file 12913_2023_10478_MOESM3_ESM.docx]

| **NPT Construct** | **Description** | **Key Qualitative Findings** |
| --- | --- | --- |
| **Implementation Context** | | |
| **Strategic intentions** | Health needs and strategic objectives of the NG-LFA at primary healthcare level | STI control and testing needs included limitations with syndromic screening, the lack of etiological management and treatment failure |
| **Negotiation capacity** | Descriptions of the current work environment to inform adaptation | Healthcare workers described team compositions and working alongside government clinic staff for the identification of patients. Further healthcare workers gave examples of patients that were referred with recurring symptoms |
| **Adaptive Executions** | Identified departments and target populations for integrated STI POCT | Healthcare workers identified HIV testing, vitals, physical examinations as point of entry for STI screening. The clinic context could inform different workarounds for integration (e.g. vertical versus integrated care) |
| **Implementation Mechanisms** | | |
| **Coherence**  Subconstructs: *Differentiation; internalization* | The constructed purpose of the NG-LFA and how it compares to other screening services | Implementation staff described how familiarity with other rapid tests and the ability to observe the testing process for healthcare providers and patients could promote satisfaction and trust. |
| **Cognitive participation**  Subconstructs: *Enrolment*  **Collection action**  Subconstructs: *Contextual integration* | Descriptions of the NG-LFA patient testing flow including compatibility in the current work environment | Healthcare workers described team compositions and usability of NG-LFA by non-clinical staff and nurse-led symptom evaluation that could inform task-shifting/remove dependence on nurses for execution |
| **Reflexive monitoring**  Subconstructs: *Communal appraisal; reconfiguration* | How the NG-LFA is collectively assessed as worthwhile and recommendations for environment modifications for sustained use | ‘Normalization’ of STI testing may be encouraged through quick-turn around of results and improved clinical decision-making/treatment guidance that in turn reduce testing apprehension and increase knowledge of STI types |

**Additional Table 2. NPT Constructs as defined for the implementation of the NG-LFA**
